# A Low Cost IoT Cyber-Physical System for Vehicle and Pedestrian Tracking in a Smart Campus

**DOI:** 10.3390/s22176585

**Published:** 2022-08-31

**Authors:** Jamal Toutouh, Enrique Alba

**Affiliations:** Departamento de Lenguajes y Ciencias de la Computación, Universidad de Málaga, 29071 Malaga, Spain

**Keywords:** smart mobility, IoT, noise pollution

## Abstract

Human tracking and traffic monitoring systems are required to build advanced intelligent, innovative mobility services. In this study, we introduce an IoT system based on low-cost hardware that has been installed on the campus of the University of Malaga, in Spain. The sensors gather smart wireless devices (Bluetooth and Wi-Fi) anonymous information and environmental noise level around them. This research studies the spatio-temporal behavior of people and noise pollution in the campus as a short-scale Smart City, i.e., a Smart Campus. Applying specific machine learning algorithms, we have analyzed two months of captured data (61 days). The main findings from the analysis show that most university community members move through the campus at similar hours, generating congestion problems. In addition, the campus suffers from acoustic pollution according to regulations; therefore, we conclude that the proposed system is useful for gathering helpful information for the university community members and managers. Thanks to its low cost, it can be easily extended and even used in other similar environments, allowing democratic access to Smart City services as an excellent added value.

## 1. Introduction

Today, it is a fact that most of the world’s population lives in cities. This high concentration of people and vehicles in specific geographic zones has amplified the importance of issues related to the mobility of the inhabitants and the transportation of goods. In this context, smart city initiatives have emerged [1,2,3]. One of their main aims is to address mobility-centric challenges by applying smart mobility solutions [4,5,6]. Most of the proposed smart mobility services are supported by intelligent systems responsible for detecting, predicting, and efficiently managing road traffic resources. For example, a potential solution to road congestion problems (which increase travel times and greenhouse emissions) may be provided by a smart traffic lights management system that reduces the waiting time at traffic lights and improves efficient driving.

These intelligent systems that are applied to mobility challenges require gathering accurate traffic/mobility data continuously to operate, which is still a big challenge today. Essential valuable mobility data, such as the number of cars per street, the origin/destination (OD) matrices of the city, and pedestrian behavior, are scarce and difficult to obtain. Nowadays, the most common systems of obtaining this type of data only perform vehicle count or provide an estimate of the road traffic density in a given area; thus, the most widely used means of assessing mobility at present in our cities are: *(a)* traditional measurement on-road hardware (e.g., loop detectors) [7], which is expensive, traumatic to install, scarcely available, and only for vehicle detection; *(b)* cameras, which are also expensive and the accuracy of the gathered data may depend on external factors as the meteorology [8]; *(c)* floating cars [9].

Moreover, the use of cameras to track the movement of vehicles and people requires the extraction and identification of vehicle license plates and a feature vector of the person’s face/body, respectively. These data are stored and processed in the system. As these data can be used directly to identify vehicles (and their drivers) and persons uniquely, it can lead to an invasion of user privacy and provoke General Data Protection Regulation (GDPR) issues.

In recent years, with the proliferation of smart mobile devices (e.g., cellphones), another manner to obtain vehicles/pedestrians mobility information is by using the cellphone location information services [10,11,12]. Thus, in order to share the location, the user must activate a service (or application) in the cellphone to exchange the information retrieved by the Global Positioning System (GPS) module. The main benefit of these GPS-based systems is that it does not require the installation of sensors or cameras. The main issues of this approach are that: *(a)* the service used to exchange the GPS position consumes a substantial amount of energy [11] and *(b)* the service generally shares private information with a third party, usually a private entity, that manages the service [12].

A promising alternative to gather vehicles/pedestrians mobility is the installation of sensors able to detect Bluetooth (BT) signals and identifying vehicles and pedestrians by the Media Access Control (MAC) hardware addresses of their smart devices [13]. Thus, the sensors detect vehicles and pedestrians by capturing the wave signals generated by the smart devices that the people carry, e.g., cellphones and watches, or are located inside the vehicles, e.g., on-board units. This choice is experiencing fast development since it is very cheap and easy to maintain; however, the provided accuracy is limited by the low signal range of BT devices and the market penetration of the target BT devices. A previous study estimated BT penetration between 27% and 29% [14].

The main aim of this article is to propose a low-cost alternative to loop detectors, cameras, and GPS-based systems to gather vehicle/pedestrians’ mobility. Thus, a novel cyber-physical system is presented. Our system extends BT-detection-based sensors by also gathering Wi-Fi (IEEE 802.11 wireless-based) signals in order to mitigate the inaccuracy of BT detectors [15]. The idea of also detecting Wi-Fi signals is encouraged by the fact that more and more car manufacturers include wireless access points as part of the equipment of their cars. In addition, we also include a noise sensor to monitor and obtain traffic-related acoustic pollution. Thus, we present an innovative road traffic monitoring approach that combines information sensed from different sources (BT, Wi-Fi, and noise) to improve the accuracy of the measurements and, therefore, the information about the road network status.

Thus, using the proposed ad hoc cyber-physical system to track mobility, we deployed a wireless sensor network (WSN) at the campus of the University of Malaga to analyze its suitability. The installation of this infrastructure is part of the Smart-Campus initiative of the University of Malaga, see University of Malaga Smart-Campus website—https://www.uma.es/smart-campus (accessed on 30 June 2022). Smart campus initiatives aim to apply intelligent systems to provide advanced services to the university community members [16,17,18].

The remainder of this paper is organized as follows: Section 2 outlines the related work. Section 3 describes the data gathered by the sensors and the main operation to extract the information about human tracking and ambient noise. Section 4 introduces the main components of the IoT system proposed in this article. Section 5 introduces the use case at the University of Malaga applied to evaluate our system. Section 6 presents the numerical evaluation and the analysis of the data extracted. Finally, Section 7 discusses the conclusions drawn from this study and outlines the main lines of future work.

### Relevance and Novelty of the Presented Research

This subsection further describes the main aims of the research published in this article, explains how the work is structured, and discusses the relevance of this article in the scope of the journal.

The main aims of this article are:

- To detail a new IoT cyber-physical system for vehicles and pedestrians’ movement tracking based on a previously presented IoT system that only counts the number of vehicles at a given point [5]. The main advantage of our cyber-physical system presented in this article is that it aggregates data from several sensors to allow vehicle/people movement tracking and not only counting. Note that the data collected and processed can be further used to predict the movement of vehicles and pedestrians if some deep learning or machine learning algorithms are used. Using such algorithms will create a more dynamic application of the proposed system (although this article does not address this matter).
- To propose a case study to show the functionality of the cyber-physical system in the Smart Campus context. The case study analyzes vehicle/pedestrian mobility within the campus of the University of Malaga, although the use of the sensor can be easily adapted to be used on any other campus. This use case is relevant because high road traffic density situations and crowded (high-people density) areas cause discomfort to the university community members. In addition, it is important because since the COVID-19 pandemic is being experienced, university managers are trying to measure the occupation of enclosed spaces, such as classrooms, cafeterias, libraries, etc., and the movement of people; thus, it is possible to identify where (which areas) and when (at what times) are crowded. In this way, the university decision-makers can take the appropriate measures to mitigate the accumulation of people in order to avoid mass transmission of the virus among members of the university community.
- To evaluate the system’s ability to assess ambient noise and to discuss the importance of providing such information to university managers.

In order to further understand the main contributions of this work, the manuscript first introduces what data the sensors collect and how these data are collected and processed (see Section 3); second, it presents the main components of the IoT cyber-physical system proposed and the principal operation (see Section 4); third, it describes the use case applied here to show the applicability of the proposed system in the context of Smart Campus (see Section 5).

The relevance of this article in the field of IoT for Smart Cities is that it presents a sensor-based cyber-physical system for tracking vehicles and people based on simple, modular, scalable, and low-cost hardware. In addition, on top of the tracking systems based on cameras, our approach has the advantage that it does not transmit or store private information such as vehicle license plates or images (or facial/body features) of people, thus mitigating possible GDPR issues.

Furthermore, an actual use case is presented at the University of Malaga (easily adaptable to other campuses or environments) that employs the proposed system in the context of Smart Campus to evaluate the movement of vehicles and people and to measure the noise within the university campus. University managers use the information provided by the system to assess road traffic, mobility of university community members, and environmental noise levels.

## 2. Related Work

Researchers and Smart City practitioners have proposed different approaches to obtain information about the inhabitants’ mobility in different contexts and on various scales. Traditionally, this information has been obtained by different methods, such as collecting travel diaries and surveys [19]. Even though this type of methodology is able to provide accurate information, it suffers from different drawbacks, e.g., *(i)* the random sampling is impracticable and it limits the representativeness of the results; *(ii)* the highly gathering costs; *(iii)* they are usefulness from obtaining real-time data.

This study focuses on methods that rely on gathering wireless signals for monitoring mobility because they can obtain accurate real-time information, do not involve high installation costs, and are not road intrusive. The most promising approaches based on this idea use Wi-Fi, BT, and Radio Frequency Identification (RFID).

RFID technologies have been applied principally for tracking humans in indoor environments [20]. Its main advantage is its high positional accuracy; however, the use of RFID in large-scale outdoor environments is limited because it needs the users to carry an RFID tag to be detected, which critically reduces the generalization. In turn, the RFID is based on low-range communications, which reduces the detection of devices at a certain distance or moving at a relatively high speed. Due to these issues, RFID has not been considered to be used in the cyber-physical system proposed.

Several researchers have used BT and Wi-Fi signals (or beacons) to track spatiotemporal behavior in various domains and environments [21,22]; however, most of the existing research studies use just one of them [23,24,25]. The main advantage of combining the use of BT and Wi-Fi is that most inhabitants are already equipped with smart devices that use these wireless technologies, and therefore, it eases their non-participatory tracking.

The methodology of detecting and tracking devices (pedestrians and vehicles) using BT and Wi-Fi is very similar. It consists of continuously gathering wireless beacons using scanners installed at a set of given locations of interest. These scanners register every detection by storing the unique MAC address of the beacon’s source device and the precise detection time. This approach is based on the proximity principle [26], i.e., it is considered that the tracked device is located very close to or at the same point as the scanner; therefore, the path traveled by a given device (carried by a vehicle or a pedestrian) is computed by combining the information of all scanners using some data analysis method to aggregate the locations of each sensor (scanner).

BT scanners have been applied to study the tourists’ behavior in a city [25], to evaluate the users’ movement in an airport [24], at outdoor festivals [27], among other uses [26]. Lately, under the coronavirus outbreak (COVID-19), contact tracing applications have emerged to identify people in contact with an infected person [28]. These applications installed in the mobile devices collect BT MAC addresses of the nearby devices (i.e., act as scanners) to share the information with governmental institutions [29]. All these approaches identify each user individually by registering their devices’ MAC address, and they provide very competitive results; however, the detection ratio depends on the operational state of the BT device. BT has three different ones: off, on-invisible, and on-visible. Only devices in on-visible mode are detectable by BT scanners. Thus, researchers presented different results in terms of detection ratio, e.g., 7% [30], 8% [25], and 11% [27]. This low detection ratio drops the sample size when tracking the population. Moreover, BT scanning suffers from the limitation that the detection range of BT is negatively affected by signal interferences [26]. These interferences are generated by other electronic devices, physical objects, etc.

Wi-Fi scan-based sensors have also been applied to extract features from a human’s spatio-temporal movement in different environments by using a similar approach to BT. The detection ratio of devices that uses Wi-Fi interfaces is more competitive (higher) than when scanning BT [21,22]. This is due to different reasons: *(i)* The users usually tend to keep their device’s Wi-Fi turned on to increase the chance of connecting to any nearby Wi-Fi network; *(ii)* The BT discovery time is ten times longer than the Wi-Fi one; and *(iii)* The Wi-Fi communication range is much longer than the BT one, and therefore, devices that are farther are detected. The more extended communication range of the Wi-Fi connections is also a disadvantage when using these communication interfaces to monitor users. The accuracy of the tracking system is limited because the device is supposed to be located at the same point as the sensor; however, as it is not designed for short-range communications such as BT, the Wi-Fi device could be located at a given significant distance.

The main difference between our approach and other systems proposed in the literature is the combination of the data coming from the detection of BT and Wi-Fi signals and the level of sound noise. The combination of these data sources provides a global knowledge of the road traffic status and people density in a given point and their mobility: the system captures MAC addresses and noise data, converts it into aggregated information, and then builds knowledge out of it.

Thus, the main advantages of the proposed system over the ones presented in the literature are:

- Compared to on-road hardware (e.g., loop detectors): Our sensors consist of simple hardware devices, which are easy to find in the market, low-cost, and easy to install and maintain (i.e., do not require road works). The sensors consume low energy (i.e., they can be fed with solar cells). In addition, our cyber-physical system detects vehicles and people. In general, on-road hardware gathers information only about vehicles.
- Compared to cameras: Our system does not transmit or store images or data of vehicle license plates or pedestrian faces/bodies; thus, it mitigates possible GDPR issues. Furthermore, the systems based on cameras apply high computationally cost algorithms to process the images and obtain the data. In contrast, our approach uses basic methods to extract the MAC addresses from the gathered packets/datagrams.
- Compared to tracking systems based on wireless signal detection: Our proposal improves the accuracy of the other ones in the literature because we decided to simultaneously capture the signal from the two types of network interface (i.e., Bluetooth and Wi-Fi). Detecting both types of wireless signals improves the likelihood of detecting devices.
- Compared to all the other systems: In addition to the detection of the wireless device, our cyber-physical system gathers noise levels because there is a correlation between noise pollution and road traffic [31]; thus, the noise level gathered by the sensors can be used to improve the accuracy of the road traffic estimate. Moreover, it has been demonstrated that high noise level causes health issues [32,33]; therefore, it is a crucial aspect to be measured and controlled in our smart cities.

## 3. Data Collected and Processes for Smart Mobility Evaluation

The IoT cyber-physical system proposed and applied in this article measures and evaluates vehicle/pedestrian mobility by detecting and tracking smart devices and gathering environmental noise. In order to detect smart devices, the system captures the Bluetooth and Wi-Fi signals produced by the smart devices’ wireless interfaces and extracts their MAC address. Moreover, the system processes the MAC address gathered data to track smart devices (i.e., evaluate their mobility).

This section details the data captured and the processes carried out by our sensors to detect smart devices, track movement, and measure environmental noise.

### 3.1. Smart Devices Detection

The proposed sensors detect smart devices by extracting the information captured in the packets that the devices broadcast throughout their wireless interfaces (Bluetooth and Wi-Fi).

In order to detect Bluetooth signals generated by smart devices, the Bluetooth interface of the sensor is set to discovering mode [34]; thus, the sensor is able to capture the packets that the nearby smart devices with Bluetooth interface enabled are broadcasting. After obtaining the Bluetooth packet, the sensor applies a process to extract the MAC address information to identify the Bluetooth interface of a given smart device and, therefore, to identify the smart device itself.

As Bluetooth communication range is limited and users of smart devices may disable the Bluetooth interface, the designed sensor gathers information from Wi-Fi signals in order to maximize the likelihood of detecting the devices. Smart devices that apply 802.11-based protocols continuously broadcast packets containing the MAC frame. The information encapsulated in the frame is useful for tasks such as control medium access, roaming support, and authentication [35]. The Wi-Fi interface of the sensor is configured to be in promiscuous mode to capture the packets broadcasted by the smart devices. The sensor is able to capture the MAC address, which is stored in one of the fields of the MAC frame, to identify the smart device’s Wi-Fi interface.

### 3.2. Human Tracking

Human tracking is carried out using the specialized wireless interfaces installed in our sensors. As presented before, the main idea is to capture valuable information that the smart devices broadcast through their Bluetooth or Wi-Fi communication interfaces to identify them individually. Our sensors extract MAC addresses from the wireless signals. The sensor’s location can be used as an approximation of the location of the detected device. Storing this information with the exact time when the device was detected (time stamp) allows the system to detect human flows.

**Figure 1:**
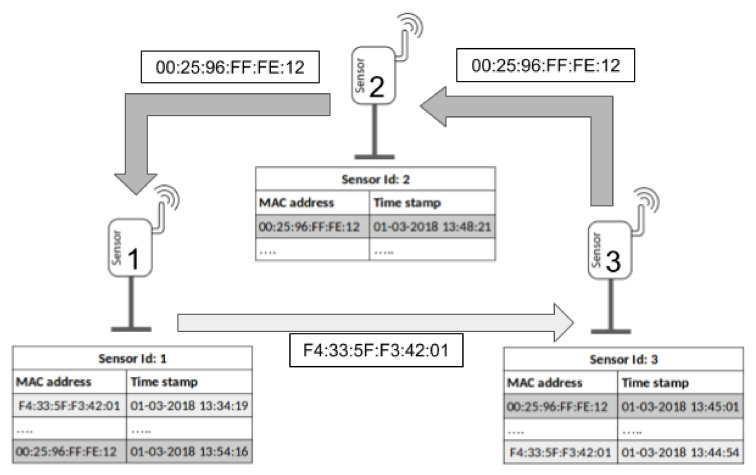
Global view of the WSN.

Therefore, in our study, the movement of a given device from the location of the sensor $s_{i}$ to the location of the sensor $s_{j}$ is detected when a specific MAC address is captured in $s_{i}$ in a $t_{si}$ time, and after a Δt time, the same MAC is detected in $s_{j}$, i.e., $t_{sj}=t_{si}+\Delta t$. Figure 1 illustrates an example of how the system is able to track devices, showing the data stored by each sensor. According to these data, the sensors have detected two different flows:

- Device with MAC address F4:33:5F:F3:42:01, which has moved from the location of *Sensor 1* to *Sensor 3* in 10 min and 35 s.
- Device with MAC address 00:25:96:FF:FE:12, which has moved from the location of *Sensor 3* to *Sensor 1* crossing the location of *Sensor 2* in 9 min and 15 s.

### 3.3. Environmental Noise Evaluation

The noise level data are captured by using the accurate sound meter introduced in Section 4.2. This device measures the noise level twice per second and sends the data via its USB interface. The data are stored in the local database of the sensor together with a given time stamp. Then, the sound is evaluated in terms of *equivalent sound pressure level*, i.e., $L_{eq}$, which expresses the mean of the sound pressure perceived by an individual measured in decibels (dB) in an interval of time [36]. In our case, $L_{eq}$ is calculated for one-minute intervals. When it is required, all these data are sent to the server, which stores it and uses it to generate the noise maps of the campus and to perform further computations.

## 4. Cyber-Physical System Design and Operation

This section describes the main components of our cyber-physical system, which is deployed as a wireless sensor network (WSN) at the university campus. The main purpose of this system is to measure the spatio-temporal dynamics of the people throughout the campus and to evaluate the environmental noise. This section first illustrates the IoT devices used to take the data and second presents the global system operation.

### 4.1. IoT Hardware Equipment

**Figure 2:**
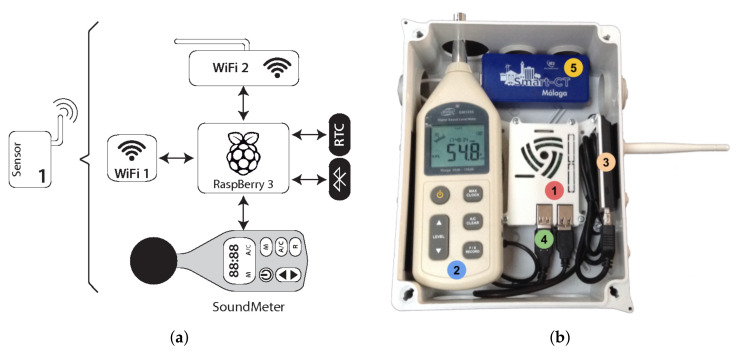
Different elements and views of the road sensor system: (**a**) Sensor block diagram; (**b**) Actual photo of the sensor.

The design of our IoT devices is mainly focused on overcoming the high costs and the sensing limitations of the traditional road traffic data collectors [7]. In order to reduce the required deployment budget, the sensors are developed by equipping them with broadly used low-cost hardware components. Table 1 presents the prices of each component. Figure 2 shows the block diagram and a real picture of the designed sensor. The sensor consists mainly of a Raspberry Pi (1), which constitutes the core of the sensor, that is equipped with a digital sound meter (2) and two external wireless interfaces: Wi-Fi (3) and Bluetooth (4).

The Raspberry Pi is a low-cost and credit-card-sized computer. It performs the required tasks for (1) individually identifying smart devices by detecting their wireless signals, (2) obtaining the sound data coming from the sound meter, (3) synchronizing the data by adding time stamps, (4) temporally storing and filtering the data, and (5) interacting with the server using wireless connectivity.

The sound meter installed in our sensors is the *BENE TECH GM1356*, see BENE TECH website—http://www.benetechco.net/en/products/gm1356.html (accessed on 30 June 2022), which uses a USB interface to send the noise level values to the Raspberry Pi. This meter is able to measure noise levels in the range between 30 and 130 decibels (dB). The external wireless interfaces used in our sensor (the *Wi-Fi TP-LINK TL-WN722N*, see TP-LINK website—https://www.tp-link.com/en/home-networking/high-gain-adapter/tl-wn722n/ (accessed on 30 June 2022), and the *Bluetooth ASUS USB-BT400*, see ASUS website—https://www.asus.com/Networking-IoT-Servers/Adapters/All-series/USBBT400/ (accessed on 30 June 2022), adapters) have been selected taking into account that they can be used in *monitor mode* to extract the MAC addresses of the detected devices.

### 4.2. Global System Operation

**Figure 3:**
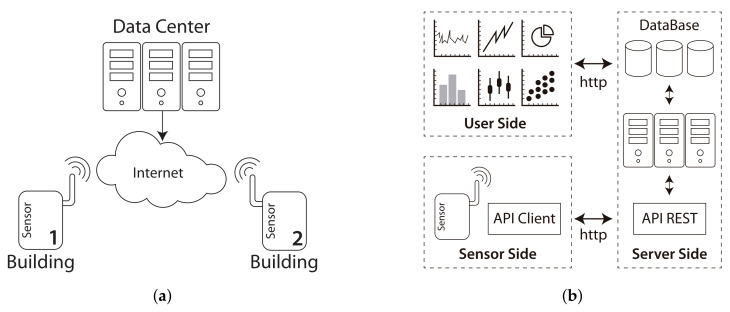
Communication and data flow of our system: (**a**) Global view of the WSN; (**b**) basic data flow of the system.

Our IoT devices are connected to a WSN whose global architecture is shown in Figure 3a. The sensors use their wireless interfaces to connect to the Internet in order to communicate with our data center. This communication has two different proposes, first, gathering data from the sensors periodically, and second, performing maintenance tasks on the sensors.

In order to carry out the transfer of data from the sensors to the data center, an architecture based on the client/server model was designed (see Figure 3b), A set of web services have been developed to establish the protocols for correctly exchanging data and instructions.

An application protocol has been developed to determine the sensors’ status. This protocol defines a procedure in which each sensor sends a packet continuously (every minute) to the server, including information about its current status. In turn, the sensors are equipped with a self-protection method, which allows automatically restoring the sensor in case of failure.

Finally, a server performs three main tasks in our data center: (1) storing the data in a database, (2) applying different methods to generate information and knowledge from the data, and (3) providing a web interface to visualize the key information.

## 5. Evaluating Vehicles and People Mobility on the Campus of the University of Malaga

In this article, we have defined a use case to evaluate the IoT system proposed at the University of Malaga. Note that our system can be easily installed and adapted to be used over any other university campus or area.

**Figure 4:**
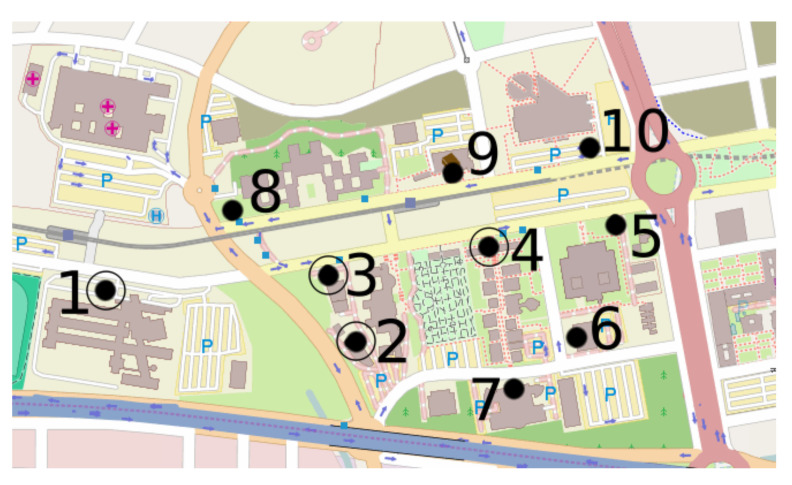
Location of the sensors installed in the Campus of Teatinos.

In this study, we have made use of the infrastructure installed in the Campus of Teatinos of the University of Malaga, that covers an area of 0.71 Km^2^. This infrastructure consists of ten sensors, as the ones introduced in Section 4, installed throughout the whole campus and a server to store and perform the main processes. In Figure 4, the black dots point out the location of the sensors. These locations in strategic places were selected in order to measure the flows of people through the campus and to generate OD matrices. In turn, the sensors are in the main entrance of the faculties to be able to measure the affluence of people to them.

The dots marked with a circle are the four sensors that gathered the data used in this study. We have selected the data from these four sensors because they are the ones that have been operating longer, and therefore, they provided more data. The data analyzed cover two months (from 9 January to 9 March of 2019), i.e., the data evaluated were captured during 61 days.

This article is focused on extracting information about the human movement behavior and the environmental noise generated through the campus. It is important to remark that, as the study takes place on a university campus, most human movements are carried out during working days (from Monday to Friday) and regular university school opening hours (from 8:00 h to 21:00 h); therefore, the analysis of the human spatio-temporal patterns (human flows) proposed here takes into account these periods of time.

In order to improve the knowledge about the evolution of the environmental noise on the campus throughout the time, we analyzed the level of noise sensed by the four sensors studied.

The proposed use case is relevant because the university managers at the University of Malaga are concerned about (a) the number of users in some specific university facilities (e.g., library and cafeteria) because crowded areas provoke discomfort; (b) the mobility of the university community members because traffic jams negatively affect the daily life of the university community members (e.g., students arrive late to their classes); (c) the noise levels suffered by the university community members may negatively affect to the running of the classes and may provoke health issues.

In addition, this use case is relevant because since the COVID-19 pandemic is being experienced, university managers are trying to measure the occupation of enclosed spaces such as classrooms, cafeterias, and libraries, in order to control the virus transmission among members of the university community.

Therefore, the information provided by the IoT system proposed here can be used by the university managers to scale services and change their schedules to avoid overcrowding in facilities such as cafeterias or libraries. Moreover, the managers could select different opening and closing times for several schools to decrease the transit of people through the campus during similar hours. In turn, the managers could avoid placing classes in the noisiest areas of the campus so that students do not suffer from high noise levels.

## 6. Experimental Analysis

This section presents the experimental analysis carried out to evaluate the human movement and environmental noise at the campus.

First, we discuss the number of smart devices detected by each sensor in order to evaluate affluence of people in a given area. Then, we analyze different spatio-temporal aspects of the tracked human flows by using our cyber-physical system. Finally, we study the environmental noise captured on the campus.

### 6.1. Counting People in a Given Area

In order to measure the number of smart devices identified by our sensors, the number of different MAC addresses captured by each sensor during a given period of time (in this case ten minutes) is evaluated. As the sensors detect no moving devices (e.g., wireless routers, desktop computers), a filtering pre-process of the data is applied in order to discard the MAC addresses of such static devices in further analyses.

**Figure 5:**
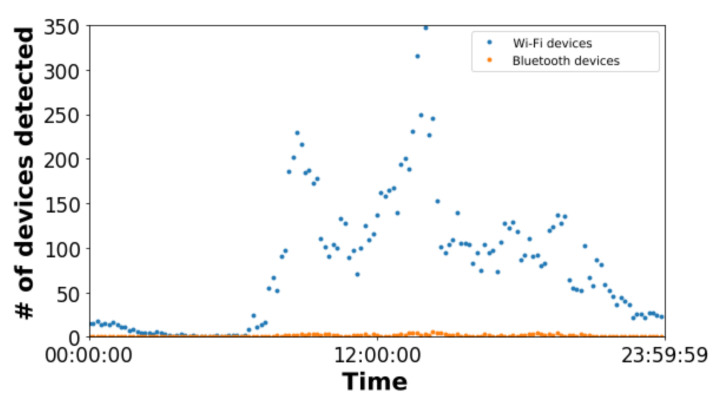
Average value of the number (#) of devices detected for both Bluetooth and Wi-Fi devices.

Table 2 shows the total number of detected devices averaging them in terms of day: Mon (Monday), Tues (Tuesday), Wed (Wednesday), Thurs (Thursday), Fri (Friday), Sat (Saturday), and Sun & Holi (Sunday and holiday), and detector: type Wi-Fi and Bluetooth. Figure 5 illustrates the average number of devices captured for all the studied sensors along the day grouping the results depending on the wireless technology used. According to the results in Table 2 and Figure 5, the number of Bluetooth devices detected is negligible compared to the number of Wi-Fi ones.

These results confirmed most smartphones have the Wi-Fi interface enabled but not Bluetooth one. In turn, the communication range of Wi-Fi communications is higher than Bluetooth communications. This allowed the sensors to detect more Wi-Fi devices (at longer distances), however, this may lead to have less accurate results since these devices may be very far from the sensor (that determines their location).

**Figure 6:**
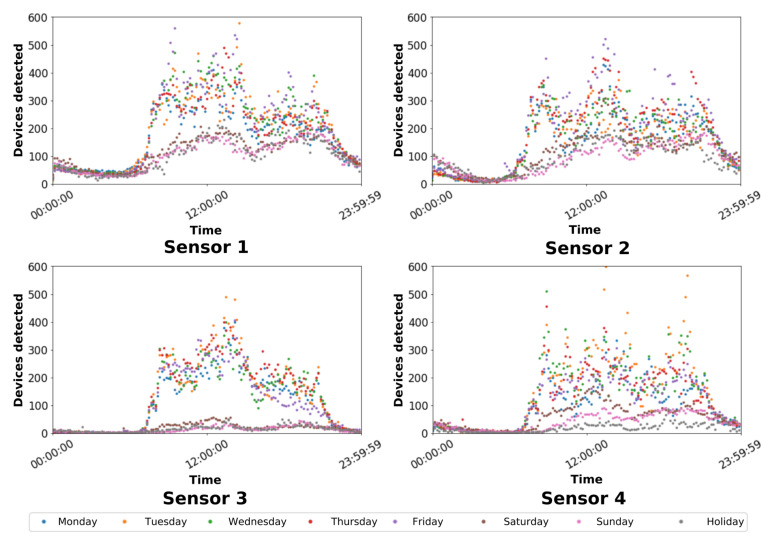
Number of devices detected over the day grouped by the week days and holidays.

Useful findings can be drawn about people’s behavior by analyzing the patterns of people flows into the buildings. In our study, our sensors are installed nearby the main doors of the buildings to gather data about people crossing throughout the campus streets, but also to provide the detection of the people entering or leaving the facilities. Thus, we evaluate the devices sensed by each sensor to extract affluence patterns. Figure 6 and Table 3 summarize the results by averaging the number of sensed devices.

The results shown in Figure 6 present three main general patterns:

- **Late-night shift** (every day from 0:00 h to 7:00 h): Device detection was almost negligible; therefore, there were hardly any people moving around in the different facilities/buildings on campus.
- **Standard working days** (from Monday to Friday; from 7:00 h to midnight): The number of detected devices increased dramatically from 7:00 h. Three peaks were able to be observed in which the sensors detected more people. The most prominent is around 14:00 h in the afternoon. In turn, there were two detection peaks that were less pronounced, one around 9:00 h and another around 21:00 h. These peaks coincide with the times when students enter and leave their classes.
- **Standard non-working days** (Saturdays, Sundays, and holidays; from 7:00 h to midnight): It was a similar pattern to the one shown above (i.e., standard working days), but far fewer people were detected. These days the buildings are mainly staffed by workers performing maintenance or research tasks.

As it was observed, in general, the flow into the buildings depends on the working and studying times at the university campus.

Table 3 presents the average of the total amount of devices detected in terms of sensor id: Sensor 1, Sensor 2, Sensor 3, and Sensor 4; type of day: Mon (Monday), Tues (Tuesday), Wed (Wednesday), Thurs (Thursday), Fri (Friday), Sat (Saturday), Sun (Sunday), and & Holi (holiday). In turn, this table shows the mean and the normalized standard deviation for each day of the week, i.e., Mean day and Stdev day (%), and each sensor, i.e., Mean sen and Stdev sen (%).

The Mean day results in Table 3 confirm the main findings of the patterns discussed above. The number of sensed devices is significantly higher during the standard working days (from Monday to Friday) than during Saturdays, Sundays, and holidays. Friday was when the mean value of the amount of sensed devices was the highest (22,877). The lowest affluence on the university campus buildings was sensed during holidays (only 8343 devices were detected).

Focusing on the people affluence in the buildings, i.e., sensor ids in Table 3, the building with the largest affluence of people was detected in the Computer Science School (i.e., Sensor 1) with an average of 23,324 sensed devices. This result is mainly due to the Computer Science School being the school with the largest amount of students and professors among the four faculties on the campus studied in this research.

Sensor 3 and Sensor 4 were installed in office buildings used for administrative and research work. Thus, the affluence of students in these buildings was limited. For this reason, the number of sensed devices was lower than for the other two sensors. In addition, the location of these two sensors has to be taken into account to evaluate their results. They were installed on the walls of these buildings separated from the roads and the sidewalks (see the map in Figure 4). This relatively long distance from the target devices made it difficult to detect the devices, provoking the high standard deviation results (see Stdev sen (%) in Table 3) and the very scattered results shown in Figure 6.

### 6.2. Tracking Human Flows

As working days (from Monday to Friday) represented the days with the highest people flow throughout the campus, this section analyzes the human spatio-temporal patterns (human flows) during these days. Thus, Four different movements or flows of humans (devices) were tracked. They are named: Flow A, movement from Sensor 2 to Sensor 3; Flow B, movement from Sensor 3 to Sensor 2; Flow C, movement from Sensor 3 to Sensor 4; Flow D, movement from Sensor 4 to Sensor 3.

**Figure 7:**
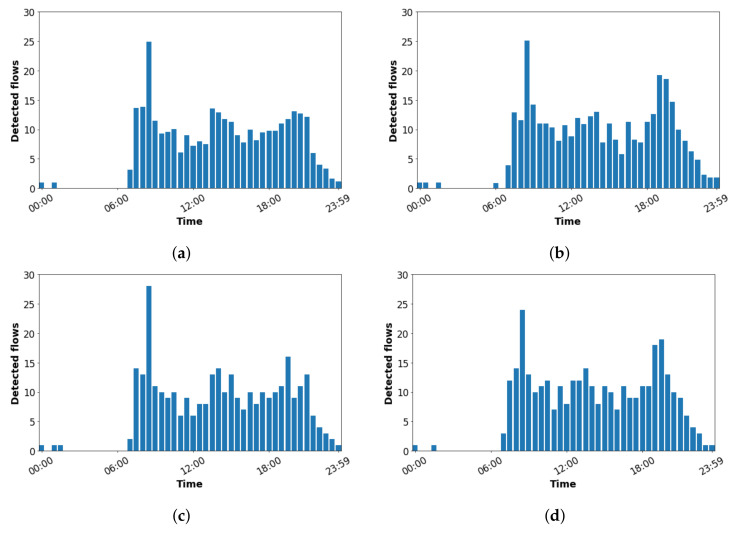
Mean number of detected flows over the day during working days. (**a**) Flow A: from Sensor 2 to Sensor 3; (**b**) Flow B: from Sensor 3 to Sensor 2; (**c**) Flow C: from Sensor 3 to Sensor 4; (**d**) Flow D: from Sensor 4 to Sensor 3.

The bar diagrams in Figure 7 show a similar distribution for the four evaluated flows. During late-night hours, the sensors detected hardly any flows. Almost all flows were tracked during regular working hours. The highest number of flows were tracked during the school starting hours (i.e., between 8:30 h and 9:00 h). In turn, Figure 7 presents two other marked peaks: the first peak happens at around 14:00 h and the second peak around 19:30 h, corresponding to school end times of the teaching for the morning and afternoon shifts, respectively.

In general, the human tracking flows results are in line with the results about the number of detected devices presented in Section 6.1, i.e., the times in which the sensors sensed the maximum number of devices are the same ones in which the system tracked the highest number of flows.

During the schools opening times (i.e., between 8:30 h and 9:00 h), there were detection of a significant number of flows. although there were less evident increases during the end of school hours (14:00 h and 19:30 h).

The high rise of human flows tracked during the schools opening times (i.e., between 8:30 h and 9:00 h) and the less evident increases during the end of school hours (14:00 h and 19:30 h) is mainly due to a remarkable number of students that arrived in the morning staying at the campus until late afternoon (these students might be attending afternoon shift classes or visiting the libraries). Generally, these students eat in the university cafeterias on campus.

In addition, our cyber-physical system allows the evaluation of the speed of the tracked flows. The average speed of the human flows gathered was 2.95 m/s (10.62 km/h), representing a relatively high pace for human walking. This is mainly due to the system not only tracking people walking but also people using vehicles to move through the campus, such as bicycles, motorcycles, and cars. Thus, the highest speed of a tracked flow was 13.1 m/s (47.2 km/h), which would correspond to a motor vehicle, such as a car.

The insights provided by analyzing the sensed devices and the human flows tracked by our cyber-physical system directly assist the university managers. We have observed that choosing the same opening and closing hours for several university schools provoked high mobility of people at similar times, which may produce road congestion and overcrowding issues. The university managers could select different opening and closing times for several schools in order to decrease the transit of people during similar hours.

### 6.3. Evaluating Noise Level

**Figure 8:**
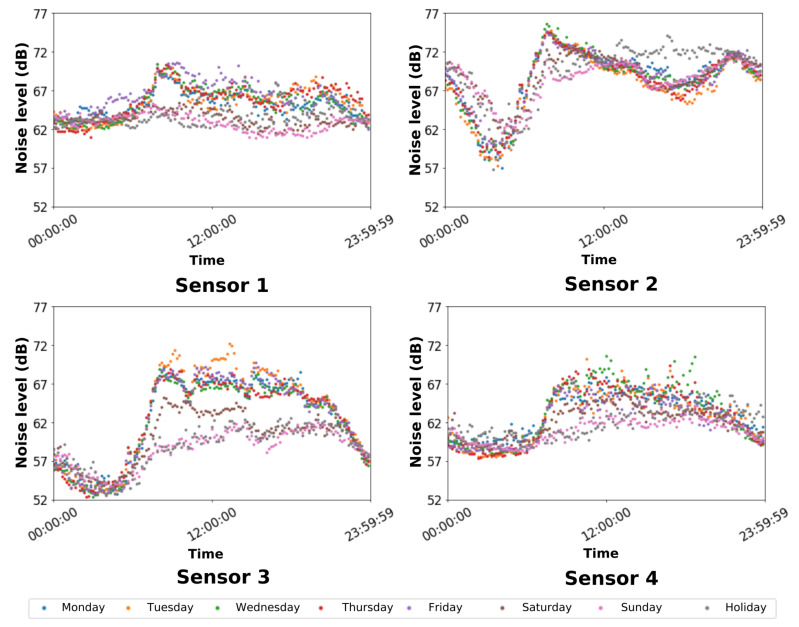
Average noise level sensed over the day grouped by the week days and holidays.

As in Section 6.1, the results were grouped each time minutes. In this case, we studied the average noise level every ten minutes in terms of dBs. Figure 8 illustrates the distribution of the levels of noise groped by type of day.

In this case, the patterns defined by the results in Figure 8 do not follow similar patterns. Even, they present some similar general behavior:

- The times with the lowest noise levels for the four sensors are the ones between midnight and 7:00 h, which coincides with the hours in which we sensed the lowest number of smart devices.
- The level of noise increases critically from 7:00 h. This increment depends on the type of the day: it is higher for working days than for no working days; however, these differences are less marked than in the data of the number of detected devices and human flows (see Section 3.2 and Section 6.1).
- Sensor 1 and Sensor 4 results do not present significant differences regarding the different type of days or times. This is mainly because they are installed close to some continuous noise source.

**Figure 9:**
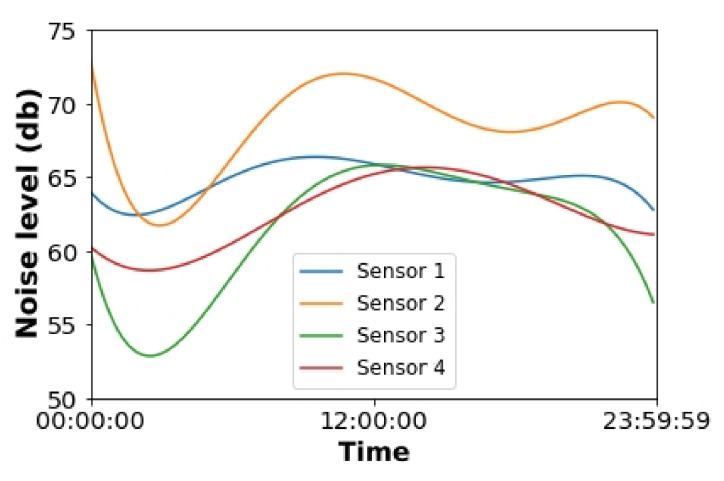
Polynomial curve fitting of the levels of noise for each sensor.

In order to evaluate the noise level depending on the sensor, we have applied a polynomial curve fitting to the points defined by the distribution of the noise level measures. Thus, we have obtained a curve that describes the noise evolution throughout the day. Figure 9 shows the computed curves.

As it can be shown in Figure 9, the maxima and minima presented by these curves of noise appear during the same periods as the same number of devices and flows detected (see Section 6.1 and Section 6.2); therefore, we can conclude that the primary source of noise on the campus is the movement of people through the campus (i.e., road traffic).

According to the results in Figure 9, Sensor 1 measured the highest levels of sound. Moreover, an essential insight of this study was that the location of the four sensors suffers from acoustic pollution according to the Malaga city council regulations [37], since the noise levels are higher than 50 dB during the night and higher than 60 dB during the day.

The university managers should consider these results and the benefit of maintaining an infrastructure of sensors to evaluate noise since noise pollution is a common unpleasant problem that negatively affects human behavior and health; thus, the European Commission adopted a directive in which the institutions are required to gather real-world data on noise exposure to promote local actions to mitigate possible issues [32].

## 7. Conclusions and Future Work

This study presents an IoT cyber-physical system based on a WSN installed at the Campus of Teatinos of the University of Malaga to capture human mobility through the campus and the environmental noise, which are two critical factors affecting the quality of life of the members of the university community. The design of the system allows expanding the sensors with new components to evaluate other metrics (e.g., temperature and humidity). Moreover, the whole methodology proposed here can be easily adapted to be used on any other campus. The main results show that accurate information can be captured.

According to the evaluation of the data captured by our system over two months, the patterns of the collected data about the movement of people are very similar to the same noise levels measured. For example, the time that obtained the highest number of tracked human flows matches with the highest amount of measured noise, which corresponded to the regular university school starting hours. In turn, these patterns may be classified into two main groups: working days and not working days.

Regarding the human movement, the data demonstrated that most students across the university community move through the campus at similar hours (at 8:30 h, 14:00 h, and 19:30 h). This generates most of the congestion problems at the campus, but they could be mitigated by shifting the scholar schedules.

The evaluation of the noise levels sensed resulted in the campus suffering from acoustic pollution according to the Malaga city council regulations since the noise levels are always higher than the ones set by this organism.

The analyses presented in this study are helpful for the university managers since they pointed at critical aspects that could be improved to benefit the whole university community. It has been observed that most mobility through the campus occurs at similar times, which may produce road congestion and overcrowding issues. Moreover, it has been seen that university community members suffer from high acoustic pollution at the location of the sensors; thus, the university managers have to take measures to change the spatio-temporal behavior of the university community members (e.g., changing school schedules) and to allow the community members to avoid areas of the campus with high noise levels.

The main lines for future work are focused on four main aspects: first, proposing a scalability analysis to evaluate our cyber-physical system on other dimensions by including data from more sensors and longer times; second, applying machine learning and deep learning methods (e.g., clustering, regression, and neural networks) to provide deep insights into the data and predictions; third, analyzing privacy issues and GDPR violations that the operation of our system may rise; forth, devising a website to offer information and knowledge about the university campus to the university community.

## Figures and Tables

**Table 1:** Price of the components used to deploy the sensors of the proposed IoT system.

Component	Price
Raspberry Pi 3	€34.31
Wi-Fi TP-LINK TL-WN722N	€9.99
Bluetooth ASUS USB-BT400	€9.99
BENE TECH GM1356 soundmeter	€60.02
Waterproof box	€4.49
Total cost	€118.80

**Table 2:** Average number of devices sensed per day grouped by sensor type.

Interface	Mon	Tues	Wed	Thurs	Fri	Sat	Sun & Holi
Wi-Fi	19,803.1	23,231.3	22,591.9	23,294.1	23,655.2	14,512.2	12,790.6
Bluetooth	542.8	601.2	639.9	650.2	622.1	220.1	166.2

**Table 3:** Average number of devices sensed per day grouped by sensor id and type of day. The maximum and minimum mean values are marked in red and green, respectively.

	Mon	Tues	Wed	Thurs	Fri	Sat	Sun	Holi	Mean Sen	Stdev Sen (%)
Sensor 1	24,874	28,766	29,258	26,818	30,585	16,558	14,812	14,918	23,324	27.0
Sensor 2	22,294	21,305	22,856	25,804	27,776	16,679	12,877	13,578	20,396	37.8
Sensor 3	16,519	19,224	17,637	19,784	16,336	2743	1902	2163	12,039	63.5
Sensor 4	14,505	22,087	19,844	18,533	16,809	8661	5926	2714	13,635	48.4
Mean day	19,548	22,846	22,399	22,735	22,877	11,160	8879	8343		
Stdev day (%)	21.5	15.7	19.5	15.9	27.9	52.4	58.7	71.0		

## Data Availability

Not applicable.
